# Supplementary material for: Genetic Variant ABCC1 rs45511401 Is Associated with Increased Response to Statins in Patients with Familial Hypercholesterolemia
Source: Pharmaceutics. 2022 Apr 27;14(5):944. doi: 10.3390/pharmaceutics14050944 (PMC9144204; doi:10.3390/pharmaceutics14050944)
Supplement: Supplementary file 1 [file pharmaceutics-14-00944-s001.zip › pharmaceutics-1634615-supplementary.pdf]

Supplementary material

Table S1 Panel of statin PK-related genes sequenced.

**PK-related genes**
**Drug transporters**
ABCB1
ABCB11
ABCC1
ABCC2
ABCC3
ABCG2
SLC15A1
SLC22A1
SLC22A6
SLC22A8
SLCO1B1
SLCO1B3
SLCO2B1
**Drug-metabolizing enzymes**
CYP1A2
CYP2C19
CYP2C8
CYP2C9
CYP2D6
CYP3A4
CYP3A5
UGT1A1
UGT1A3
UGT2B7

Table S2 Clinical data of FH patients classified according to CAD risk.

		Total (n=114)	CAD risk[a] Very high (n = 64)	High (n = 11)	Intermediate (n = 39)	*p value*
**High risk factors**						
Gender	Male	28.1 (32)	34.4% (22)	36.4% (4)	15.4% (6)	*0.093*
Medical history[b], %	AMI	29.2 (33)	51.6 (33)	0.0 (0)	0.0 (0)	***<0.001***
	CAD	40.0 (42)	70.0 (42)	0.0 (0)	0.0 (0)	***<0.001***
	CVE	6.0 (6)	10.3 (6)	0.0 (0)	0.0 (0)	*0.099*
	Angina	40.6 (41)	69.5 (41)	0.0 (0)	0.0 (0)	***<0.001***
	MR	30.9 (34)	54.8 (34)	0.0 (0)	0.0 (0)	***<0.001***
Tobacco smoking[b], %		14.3 (16)	19.4 (12)	27.3 (3)	2.6 (1)	***0.027***
**Therapy factors**						
LDL-c absolute target, %	<50 mg/dL	2.6 (3)	0.0 (0)	18.2 (2)	2.6 (1)	***0.002***
	<70 mg/dL	9.6 (11)	9.4 (6)	18.2 (2)	7.7 (3)	*0.578*
	< 100 mg/dL	34.2 (39)	31.2 (20)	27.3 (3)	41.0 (16)	*0.525*
LDL-c reduction ≥ 50%			81.8 (9)	48.4 (31)	46.2 (18)	*0.095*
TT reached[c], %		12.3 (14)	0.0 (0)	18.2 (2)	30.8 (12)	***<0.001***

Number of patients in brackets. Categorical variables were compared by chi-square test. AMI: acute myocardial infarction; CAD: coronary artery disease; CVE: cerebrovascular event; LDL-c: low-density lipoprotein cholesterol; MR: myocardial revascularization; TT: therapy target.

[a] The stratification of CAD risk was performed according to the Update of the Brazilian Guideline for FH (IZAR et al., 2021):

1) Very high risk: patients carrying manifested CAD (history of AMI, angina *pectoris*, previous myocardial revascularization or ischemic or transitory CVE);

2) High risk: primary prevention with baseline LDL-c > 400 mg/dL, or baseline LDL-c > 310 mg/dL with one high risk factor (tobacco smoking, male gender or HDL-c < 40 mg/dL), or baseline LDL-c > 190 mg/dL with two high risk factors;

3) Intermediate risk: Primary prevention without high risk factors.

[b] Data were not available for history of AMI (1), CAD (9), CVE (14), tobacco smoking (2).

[c] The therapy target for each risk group was the following:

1) Very high risk: LDL-c reduction ≥ 50% + on-treatment LDL-c < 50 mg/dL;

2) High risk: LDL-c reduction ≥ 50% + on-treatment LDL-c < 70 mg/dL;

3) Intermediate risk: LDL-c reduction ≥ 50% + on-treatment LDL-c < 70 mg/dL.

Table S3 Influence of lipid-lowering treatment on serum lipids of FH patients.

**Variable**		**Total** (n=114)	**RE** (n=58)	**NRE** (n=56)	***p-value***
Total cholesterol, mg/dL	Baseline	318 (216 - 420)	330 (173 - 487)	300 (247 - 353)	***0.004***
	On-treatment	197 (133 - 261)	176 (122 - 230)	230 (170 - 290)	***<0.001***
	% change	-36 (-61 - -11)	-51 (-65 - -37)	-25 (-40 - -10)	***<0.001***
	p-value	***<0.001***	***<0.001***	***<0.001***	
LDL cholesterol, mg/dL	Baseline	226 (128 - 324)	239 (100 - 378)	222 (171 - 273)	***0.005***
	On-treatment	118 (51 - 185)	96 (60 - 132)	152 (104 - 200)	***<0.001***
	% change	-51 (-81 - -21)	-62 (-76 - -48)	-32 (-50 - -13)	***<0.001***
	p-value	***<0.001***	***<0.001***	***<0.001***	
HDL cholesterol, mg/dL	Baseline	49 (35 - 63)	50 (34 - 66)	48 (35 - 61)	*0.711*
	On-treatment	47 (30 - 64)	44 (26 - 62)	48 (35 - 61)	*0.473*
	% change	0 (-26 - 26)	-6 (-31 - 19)	0 (-22 - 22)	*0.230*
	p-value	*0.619*	*0.268*	*0.546*	
Triglycerides, mg/dL	Baseline	154 (52 - 256)	150 (18 - 282)	154 (80 - 228)	*0.511*
	On-treatment	122 (43 - 201)	105 (12 - 198)	130 (53 - 207)	*0.073*
	% change	-24 (-72 - 24)	-31 (-68 - 6)	-13 (-57 - 31)	***0.003***
	p-value	***<0.001***	***<0.001***	***0.010***	

Patients with LDL-c reduction of at least 50% after statin treatment were classified as responders. Continuous variables are shown as median and interquartile range and were compared by Mann-Whitney or Wilcoxon test. . n: number of patients; HDL: high-density lipoprotein; LDL: low-density lipoprotein; RE: responder; NRE non responder.

Table S4 Concentration of laboratory variables on treatment in FH patients grouped according to statin response.

**Variable**	**Total (n=113)**	**RE (n=58)**	**NRE (n=56)**	***p-value***
Apo AI, mg/dL	147 (112 - 182)	142 (105 - 178)	153 (121 - 185)	***0.036***
Apo B, mg/dL	125 (73 - 177)	119 (75 - 163)	150 (93 - 207)	***0.007***
Glucose, mg/dL	92 (73 - 111)	89 (76 - 102)	95 (74 - 116)	***0.004***
HbA1c, %	6 (5.3 - 6.7)	6.0 (5.2 - 6.8)	5.9 (5.2 - 6.6)	*0.617*
Creatinine, mg/dL	0.8 (0.6 - 1)	0.8 (0.6 - 1)	0.7 (0.4 - 1)	*0.075*
ALT, U/L	32 (11 - 53)	32 (12 - 52)	31.5 (11.3 - 51.7)	*0.446*
AST, U/L	26 (16 - 36)	28 (17 - 39)	24.5 (15.5 - 33.5)	*0.221*
CK, U/L	91.5 (12.3 - 170.7)	94.5 (34.5 - 154.5)	88.5 (6.3 - 170.7)	*0.924*
TSH, µIU/mL	1.6 (0.1 - 3.1)	1.5 (-0.2 - 3.2)	1.7 (0.5 - 2.9)	*0.899*
T4, ng/dL	1.0 (0.8 - 1.2)	1.0 (0.8 - 1.2)	0.9 (0.7 - 1.1)	*0.680*

Patients with LDL-c reduction of at least 50% after statin treatment were classified as responders. Continuous variables are shown as median and interquartile range and were compared by Mann-Whitney test. Information on laboratory data was missing for apo AI (33 patients), apo B (33), glucose (17), HbA1c (27), creatinine (27), ALT (25), AST (25), CK (24), TSH (23) and T4 (25). n: number of patients; ALT: alanine aminotransferase; Apo AI: apolipoprotein AI; Apo B: apolipoprotein B; AST: aspartate aminotransferase; CK: creatine kinase; HbA1c: glycated hemoglobin; HDL: high-density lipoprotein; LDL: low-density lipoprotein; T4: thyroxine; TSH: thyroid-stimulating hormone; RE: responder; NRE non responder.

Table S5 Influence of the type of lipid-lowering treatments on lipid levels of FH patients (n=114).

**Variable**		**Statin intensity**			**Ezetimibe**		
		**Moderate** (n=16)	**High** (n=98)	***p-value***	**Non-users** (n=72)	**Users** (n=42)	***p-value***
**TC**	Baseline	306 (253-359)	322 (213-431)	*0.109*	304 (239-369)	333 (204-462)	*0.011*
	On-treatment	232 (160-304)	192 (125-259)	*0.011*	203 (121-285)	188 (137-239)	*0.362*
	% change	-22 (-40 – -4)	-40 (-65 – -15)	*<0.001*	-34 (-53– -15)	-47 (-74– -20)	*0.031*
	p-value	*<0.001*	*<0.001*		*<0.001*	*<0.001*	
**LDL-c**	Baseline	222 (149-295)	230 (123-337)	*0.071*	221 (169-273)	244 (142-346)	*0.001*
	On-treatment	130 (68-192)	116 (58 - 174)	*0.116*	117 (49-185)	122 (65-179)	*0.936*
	% change	-32 (-56 – -8)	-53 (-72 – -31)	*0.002*	-47 (-73–-21)	-61 (-88–-34)	*0.009*
	p-value	*<0.001*	*<0.001*		*<0.001*	*<0.001*	
**HDL-c**	Baseline	52 (44-60)	48 (33 - 63)	*0.103*	49 (34-64)	49 (34-64)	*0.342*
	On-treatment	53 (36-70)	46 (29-63)	*0.086*	45 (29-61)	48 (32-64)	*0.374*
	% change	-6 (-38 - 26)	0 (-23 - 23)	*0.003*	-1 (-26 - 24)	0 (-24 - 24)	*0.764*
	p-value	*1.000*	*0.680*		*0.837*	*0.581*	
**TG**	Baseline	162 (100-224)	154 (42 - 66)	*0.831*	157 (41-273)	142 (41-243)	*0.464*
	On-treatment	157 (73-241)	110 (34-186)	*0.004*	130 (30-230)	108 (38-178)	*0.085*
	% change	-4 (-39 - 31)	-27 (-75 - 21)	*0.001*	-24 (-68-20)	-24 (-75-27)	*0.438*
	p-value	*0.391*	*<0.001*		*<0.001*	*<0.001*	

Continuous variables are shown as median and interquartile range and were compared by Mann-Whitney test. FH: familial hypercholesterolemia; HDL-c: high-density lipoprotein cholesterol; LDL-c: low-density lipoprotein cholesterol; TC: total cholesterol; TG: triglycerides.

Table S6 Biodemographic characteristics of FH patients with SRAE (n=114).

**Variable**[a]		**Total** (114)	**No SRAE** (90)	**SRAE** (24)	*p-value*
Age, years		57.1 (37.9-76.3)	57.3 (38.3 - 76.3)	56.9 (38.9 - 74.9)	*0.830*
Gender (female), %		71.9 (82)	72.2 (65)	70.8 (17)	*1.000*
Ethnics, %	White	53.5 (54)	53.2 (41)	54.2 (13)	*0.538*
	Brown	31.7 (31)	29.9 (23)	37.5 (9)	
	Black	14.9 (15)	16.9 (13)	8.3 (2)	
Xanthomas, %		12.3 (14)	6.7 (6)	33.3 (8)	*0.002*
Arcus cornealis, %		17.9 (20)	16.9 (15)	21.7 (5)	*0.810*
FH clinical diagnosis[b], %	Definite or probable	68.4 (78)	64.4 (58)	83.3 (20)	*0.128*
	Possible	31.6 (36)	35.6 (32)	16.7 (4)	
FH molecular diagnosis, %	FH variants	30.7 (35)	24.4 (22)	54.2 (13)	*0.011*
	APOB	0.9 (1)	0.0 (0)	4.2 (1)	*0.006*
	LDLR	28.3 (32)	22.2 (20)	50.0 (12)	
	PCSK9	1.8 (2)	2.2 (2)	0.0 (0)	
	LDLRAP1	0.0 (0)	0.0 (0)	0.0 (0)	
Hypertension, %		62.5 (70)	61.4 (54)	66.7 (16)	*0.812*
Type 2 diabetes, %		21.6 (24)	18.4 (16)	33.3 (8)	*0.196*
Obesity, %		28.6 (32)	33.0 (29)	12.5 (3)	*0.087*
BMI, kg/cm²		27.7 (22.5-32.9)	27.9 (22 - 33.8)	25.9 (22.1 - 29.7)	*0.126*
Medical history, %	AMI	29.2 (33)	28.9 (26)	30.4 (7)	*1.000*
	CAD	40.0 (42)	36.5 (31)	55.0 (11)	*0.205*
	CVE	6.0 (6)	6.4 (5)	4.5 (1)	*1.000*
Alcohol consumption, %		25.0 (22)	28.8 (19)	13.6 (3)	*0.281*
Tobacco smoking, %		14.3 (16)	14.8 (13)	12.5 (3)	*0.918*
Lipid-lowering treatment, %	Atorvastatin	79.8 (91)	83.3 (75)	66.7 (16)	*0.192*
	Simvastatin	10.5 (12)	8.9 (8)	16.7 (4)	
	Rosuvastatin	9.6 (11)	7.8 (7)	16.7 (4)	
	Statins + Eze	36.8 (42)	33.3 (30)	50.0 (12)	*0.206*
Statin intensity, %	Moderate	14.0 (16)	15.6 (14)	8.3 (2)	*0.566*
	High	86.0 (98)	84.4 (76)	91.7 (22)	
Statin response	RE	50.9 (58)	42.2 (38)	83.3 (20)	*0.001*
	NRE	49.1 (56)	57.8 (52)	16.7 (4)	
Drug interactions	CYP3A4 inhibitors[c]	10 (8.8)	5.6 (5)	20.8 (5)	*0.052*
	CYP3A4 inhibitors + inducers[d]	1 (0.01)	0 (0.0)	1.0 (1.9)	-
Reduced adherence, %	Statins	15.9 (18)	10.1 (9)	37.5 (9)	*0.003*
	Ezetimibe	10.6 (12)	5.6 (5)	29.2 (7)	*0.003*

Number of patients in brackets. SRAE, group included patients that experienced all SRAE, including myalgia (19), stomach pain (4), diarrhea (1), urinary tract infection (1), increased hepatic enzymes (1) and joint pain (1). Categorical variables were compared by chi-square test. Continuous variables are shown as median and interquartile range and were compared by Mann-Whitney test. AMI: acute myocardial infarction; BMI: body mass index; CAD: coronary artery disease; CVE: cerebrovascular event; Eze: ezetimibe; NRE: non responder; RE: responder; SRAE: statin-related adverse events

[a] Data were not available for ethnics (13 patients), *arcus cornealis* (2), hypertension (2), diabetes (3), BMI (4), obesity (2), history of infarction (1), CAD (9), CVE (14), tobacco smoking (2), alcohol consumption (26), age (2). [b] DCLN modified criteria. [c] All patients in this category used the CYP3A4 inhibitor amlodipine. [d] All patients in this category used the CYP3A4 inhibitor amlodipine and the CYP3A4 inducer carbamazepine.

Table S7 Association between SRAE and serum lipids of FH patients (n=114).

**Variable**		**No SRAE** (90)	**SRAE** (24)	***p-value***
**TC**	Baseline	310 (245 - 376)	374 (239 - 509)	***0.001***
	On-treatment	192 (111 - 273)	204 (163 - 245)	***0.001***
	% change	-33.4 (-12.7 - -54.1)	-50.0 (-36.6 - -63.4)	***0.001***
	p-value	***<0.001***	***<0.001***	
**LDL-c**	Baseline	224 (169 - 279)	295 (140 - 449)	***0.007***
	On-treatment	117 (47 - 187)	121 (78 - 165)	*0.784*
	% change	-47.3 (-20.1 - -74.5)	-61.3 (-51.8 - -70.8)	***0.002***
	p-value	***<0.001***	***<0.001***	
**HDL-c**	Baseline	49 (35 - 63)	51 (30.5 - 71.5)	*0.352*
	On-treatment	47 (34 - 60)	46.5 (20.5 - 72.5)	*0.833*
	% change	0 (-26.3 - 26.3)	-6.6 (-20.9 – 7.7)	*0.325*
	p-value	*0.824*	*0.523*	
**TG**	Baseline	154 (60.8 - 247.2)	191 (41.5 - 340.5)	*0.242*
	On-treatment	119 (49 - 189)	142 (17 - 267)	*0.385*
	% change	-24.2 (-70.4 – 22.0)	-28.9 (- 91.3 – 33.5)	*0.985*
	p-value	***<0.001***	*0.279*	

Continuous variables are shown as median and interquartile range and were compared by Mann-Whitney test. FH: familial hypercholesterolemia; HDL-c: high-density lipoprotein cholesterol; LDL-c: low-density lipoprotein cholesterol; TC: total cholesterol; TG: triglycerides; SRAE: statin-related adverse events.

Table S8 Variants in PK-related genes identified in FH patients (n=114).

Gene	rs code	NT change	AA change	Type	MAF (%)	*In silico* prediction	HWE p -value
ABCB1	rs2032582	c.2677T>G	p.Ser893Ala	missense	59.2	N	*0.247*
	rs28364277	c.*146G>A		3'UTR	3.1	N	*1.000*
	rs2229107	c.3421T>A	p.Ser1141Thr	missense	1.3	N	*1.000*
	rs2235052	c.*82_*79delTTAC		3'UTR	2.2	N	*1.000*
	rs17064	c.*89A>T		3'UTR	7.9	N	*1.000*
	rs3842	c.*193A>G		3'UTR	12.3	N	*0.213*
	rs9282564	c.61A>G	p.Asn21Asp	missense	3.9	N	*1.000*
	rs3213619	c.-693T>C		5'UTR	4.8	N	*0.224*
	rs3747802	c.-113086T>C		5'UTR	0.9	N	*1.000*
	rs28364275	c.*21T>C		3'UTR	1.8	N	*1.000*
	rs28364278	c.*172_*173insGAGAGACA		3'UTR	1.8	N	*1.000*
	rs35023033	c.2005C>T	p.Arg669Cys	missense	0.4	N	*1.000*
	rs35730308	c.3322T>C	p.Trp1108Arg	missense	0.4	N	*1.000*
	rs28364274	c.3751G>A	p.Val1251Ile	missense	0.9	N	*1.000*
	rs57521326	c.3262G>A	p.Asp1088Asn	missense	0.9	N	*1.000*
	rs28364279	c.*252A>C		3'UTR	0.4	N	*1.000*
	rs28364280	c.*316G>A		3'UTR	0.4	N	*1.000*
	rs36008564	c.781A>G	p.Ile261Val	missense	0.4	N	*1.000*
ABCB11	rs2287622	c.1331T>C	p.Val444Ala	missense	58.7	N	*<0.001*
	rs473351	c.*236A>G		3'UTR	63.6	N	*0.009*
	rs495714	c.*368G>A		3'UTR	56.0	N	*0.037*
	rs496550	c.*420A>G		3'UTR	56.0	N	*0.037*
	rs11568364	c.2029A>G	p.Met677Val	missense	5.4	N	*1.000*
	rs1521808	c.3556G>A	p.Glu1186Lys	missense	0.5	N	*1.000*
	rs766285158	c.3691C>T	p.Arg1231Trp	missense	0.5	N	*1.000*
	Novel	c.*614G>A		3'UTR	0.5	N	*1.000*
	rs11568357	c.616A>G	p.Ile206Val	missense	0.5	N	*1.000*
	rs111482608	c.1636C>A	p.Gln546Lys	missense	0.5	N	*1.000*
	rs11568370	c.1774G>C	p.Glu592Gln	missense	0.5	N	*1.000*
ABCC1	rs129081	c.*801G>C		3'UTR	40.2	N	*0.034*
	rs3743527	c.*543C>T		3'UTR	21.2	N	*0.093*
	rs4148381	c.*1321_*1322insT		3'UTR	51.1	N	*0.000*
	rs8056298	c.*1385T>G		3'UTR	97.8	N	*<0.001*
	rs212090	c.*866T>A		3'UTR	40.2	N	*0.011*
	rs113264879	c.*883G>A		3'UTR	0.5	N	*1.000*
	rs16967632	c.*1645G>A		3'UTR	0.5	N	*1.000*
	rs142023064	c.*1293_*1297delGAAAA		3'UTR	2.2%	N	*1.000*
	rs150927043	c.*1759T>A		3'UTR	1.6	N	*1.000*
	rs4148381	c.*1321_*1322insTT		3'UTR	30.2	N	*0.802*
	rs212091	c.*1512T>C		3'UTR	11.4	N	*0.006*
	rs4148356	c.2168G>A	p.Arg723Gln	missense	0.5	N	*1.000*
	rs4148380	c.*1293G>A		3'UTR	4.9	N	*0.151*
	rs113328089	c.*228G>A		3'UTR	2.2	N	*1.000*
	Novel	c.*1293G>0		3'UTR	1.1	N	*1.000*
	Novel	c.66del5>C		frameshift deletion	0.5	D	*1.000*

Gene	rs code	NT change	AA change	Type	MAF (%)	*In silico* prediction	HWE p -value
	rs45511401	c.2012G>T	p.Gly671Val	missense	3.8	D	*1.000*
	rs139158420	c.*401C>T		3'UTR	0.5	N	*1.000*
	Novel	c.*1752_*1753insA		3'UTR	1.6	N	*1.000*
	rs111601005	c.*1752delA		3'UTR	2.2	N	*1.000*
	rs45492303	c.*1237G>C		3'UTR	2.2	N	*1.000*
	rs74009607	c.*443C>T		3'UTR	2.2	N	*1.000*
	rs80085493	c.*1604C>T		3'UTR	0.5	N	*1.000*
	Novel	c.*1015_*1016delGC		3'UTR	0.5	N	*1.000*
	rs8187856	g.16146576C>G		splicing	1.1	N	*1.000*
	rs146369277	c.*800C>G		3'UTR	0.5	N	*1.000*
	rs183032276	c.4154G>A	p.Arg1385Gln	missense	0.5	N	*1.000*
	rs112282109	c.1898G>A	p.Arg633Gln	missense	0.5	N	*1.000*
	rs557646879	c.-88_-75del-		5'UTR	0.5	N	*1.000*
	rs147785655	c.*1000G>A		3'UTR	0.5	N	*1.000*
	rs45569938	c.*546T>G		3'UTR	0.5	N	*1.000*
	rs13337489	c.3140G>C	p.Cys1047Ser	missense	1.1	N	*1.000*
	rs28706727	c.3436G>A	p.Val1146Ile	missense	0.5	N	*1.000*
	rs143805318	c.*1644C>T		3'UTR	0.5	N	*1.000*
	Novel	c.145T>G		missense	0.5	N	*1.000*
	rs182967563	c.*272G>A		3'UTR	0.5	N	*1.000*
	rs187769078	c.185G>A	p.Arg62Gln	missense	0.5	N	*1.000*
	rs188577026	c.*891A>G		3'UTR	0.5	N	*1.000*
	rs199815778	c.4441G>A	p.Val1481Ile	missense	0.5	N	*1.000*
ABCC2	rs2273697	c.1249G>A	p.Val417Ile	missense	16.8	N	*0.429*
	rs45441199	c.3107T>C	p.Ile1036Thr	missense	1.1	N	*1.000*
	rs927344	c.116A>T	p.Tyr39Phe	missense	98.9	N	*<0.001*
	rs17222723	c.3563T>A	p.Val1188Glu	missense	7.6	N	*0.051*
	rs8187699	c.3817A>G	p.Thr1273Ala	missense	0.5	N	*1.000*
	rs8187710	c.4544G>A	p.Cys1515Tyr	missense	9.8	N	*0.136*
	rs17222617	c.2546T>G	p.Leu849Arg	missense	1.6	N	*1.000*
	rs717620	c.-24C>T		5'UTR	17.9	N	*0.701*
	rs138578110	c.*259G>T		3'UTR	1.1	N	*1.000*
	rs8187692	c.3542G>T	p.Arg1181Leu	missense	2.7	D	*1.000*
	rs7080681	c.1058G>A	p.Arg353His	missense	2.7	N	*1.000*
	rs17216317	c.3872C>T	p.Pro1291Leu	missense	3.3	D	*1.000*
	rs72558199	c.3196C>T	p.Arg1066X	stop gain	0.5	N	*1.000*
	rs141413284	c.1860T>A	p.Asp620Glu	missense	0.5	N	*1.000*
	rs533334893	g.101552117G>A		splicing	0.5	D	*1.000*
ABCC3	rs34926034	c.202C>T	p.His68Tyr	missense	1.1	N	*1.000*
	rs141856639	c.3971G>A	p.Arg1324His	missense	1.1	D	*1.000*
	rs35999272	c.2758C>T	p.Pro920Ser	missense	2.2	N	*1.000*
	rs34346931	c.1223A>G	p.Glu408Gly	missense	0.5	N	*1.000*
	rs150601692	c.4030A>G	p.Lys1344Glu	missense	0.5	N	*1.000*
	rs11568591	c.3890G>A	p.Arg1297His	missense	6.5	D	*1.000*
	rs200779271	c.980T>C	p.Ile327Thr	missense	0.5	N	*1.000*
	rs201562834	c.871C>T	p.Arg291Trp	missense	0.5	N	*1.000*
	rs1003354	c.1580C>T	p.Thr527Met	missense	0.5	N	*1.000*

Gene	rs code	NT change	AA change	Type	MAF (%)	*In silico* prediction	HWE p -value
	rs143608762	c.694C>T	p.Arg232Trp	missense	0.5	N	*1.000*
	rs35777968	c.296G>A	p.Arg99Gln	missense	0.5	N	*1.000*
	rs139106724	c.2377G>A	p.Val793Ile	missense	1.1	N	*1.000*
	rs200413276	c.2558C>A	p.Ala853Asp	missense	0.5	N	*1.000*
	rs372683132	c.922G>A	p.Gly308Ser	missense	1.1	N	*1.000*
	rs11568584	c.2153A>T	p.Lys718Met	missense	0.5	N	*1.000*
	rs11568607	g.48745787G>A		splicing	2.2	N	*1.000*
	rs11568590	c.4094A>G	p.Gln1365Arg	missense	0.5	N	*1.000*
	rs11568608	c.1820G>A	p.Ser607Asn	missense	1.1	N	*1.000*
	rs34291385	c.2293G>C	p.Val765Leu	missense	1.1	N	*1.000*
	rs200903266	c.3401G>A	p.Arg1134Gln	missense	0.5	N	*1.000*
	rs138342952	c.*258G>C		3'UTR	1.1	N	*1.000*
	rs11568588	c.4042C>T	p.Arg1348Cys	missense	1.1	N	*1.000*
	rs148804178	c.205C>G	p.Leu69Val	missense	0.5	N	*1.000*
	rs563802547	c.*140_*141insT		3'UTR	0.5	N	*1.000*
ABCG2	rs45605536	c.1582G>A	p.Ala528Thr	missense	1.1	N	*1.000*
	rs111766106	c.-18485C>T		5'UTR	0.5	N	*1.000*
	rs45510401	c.*1964T>C		3'UTR	2.2	N	*0.026*
	rs72554040	c.-91177C>T		5'UTR	8.2	N	*0.389*
	rs1448784	c.*1066T>C		3'UTR	1.1	N	*1.000*
	rs2231142	c.421C>A	p.Gln141Lys	missense	6.5	N	*1.000*
	rs2231137	c.34G>A	p.Val12Met	missense	6.0	N	*1.000*
	rs10030206	c.*1295A>T		3'UTR	1.1	N	*1.000*
	rs115770495	c.*1726G>A		3'UTR	2.2	N	*1.000*
	rs1337337886	c.131A>G	p.Tyr44Cys	missense	0.5	N	*1.000*
	rs35965584	c.1624A>G	p.Thr542Ala	missense	0.5	N	*1.000*
	rs45630471	c.-18400A>G		5'UTR	0.5	N	*1.000*
	rs2231135	c.-18847T>C		5'UTR	1.1	N	*1.000*
	Novel	c.1453C>A		missense	0.5	N	*1.000*
	rs138606116	c.1060G>A	p.Gly354Arg	missense	0.5	N	*1.000*
	rs55927234	c.-18436C>G		5'UTR	0.5	N	*1.000*
	rs34783571	c.1858G>A	p.Asp620Asn	missense	0.5	N	*1.000*
	rs34264773	c.1758A>T	p.Lys586Asn	missense	0.5	N	*1.000*
	Novel	c.*1575T>C		3'UTR	0.5	N	*1.000*
	rs34124189	g.89053790G>A		splicing	0.5	N	*1.000*
CYP1A2	rs33923017	c.*360_*361insT		3'UTR	11.4	N	*0.595*
	rs34002060	c.*1034delT		3'UTR	15.2	N	*0.213*
	rs58661304	c.*270A>C		3'UTR	5.4	N	*0.012*
	Novel	c.*1033_*1034insT		3'UTR	6.0	N	*1.000*
	rs1288558234	g.75041241del		splicing	0.5	N	*1.000*
	rs17861157	c.894C>A	p.Ser298Arg	missense	3.3	N	*0.065*
	rs45540640	c.613T>G	p.Phe205Val	missense	0.5	N	*1.000*
	rs913188841	g.75041242C>G		splicing	0.5	N	*1.000*
	rs201763966	c.142T>G	p.Trp48Gly	missense	0.5	N	*1.000*
	Novel	c.*1035_*1036insT		3'UTR	18.5	N	*0.127*
	Novel	c.*1035delT		3'UTR	18.5	N	*0.127*
	Novel	c.*1034_*1035delTT		3'UTR	19.6	N	*0.070*

Gene	rs code	NT change	AA change	Type	MAF (%)	*In silico* prediction	HWE p -value
	Novel	c.*361_*362insT		3'UTR	12.5	N	*0.600*
	Novel	c.*361_*362insTT		3'UTR	12.5	N	*0.600*
	rs201977879	c.*361delT		3'UTR	17.9	N	*0.211*
	Novel	c.*274C>0		3'UTR	1.1	N	*1.000*
	rs11636419	c.*171A>G		3'UTR	6.5	N	*1.000*
	rs150722579	c.*292_*293insC		3'UTR	1.6	N	*1.000*
	rs17861162	c.*1324C>G		3'UTR	8.7	N	*1.000*
	rs201077484	c.*274delC		3'UTR	1.6	N	*1.000*
	rs57295890	c.*282delC		3'UTR	10.3	N	*1.000*
	rs200442208	c.*282C>A		3'UTR	2.2	N	*1.000*
	rs780737808	c.*304_*305insAT		3'UTR	1.6	N	*1.000*
	Novel	c.*1034_*1035insT		3'UTR	1.1%	N	*1.000*
	rs201443593	c.*292A>C		3'UTR	0.5	N	*1.000*
	rs56141902	c.*854G>A		3'UTR	0.5	N	*1.000*
	Novel	c.*271_*274delAAAC		3'UTR	2.7	N	*0.044*
	rs758124536	c.409C>T	p.Arg137Trp	missense	0.5	N	*1.000*
	Novel	c.*283_*284insA		3'UTR	1.1	N	*1.000*
	Novel	c.*282_*283delins0		3'UTR	1.1%	N	*1.000*
	Novel	c.*283delA		3'UTR	1.1	N	*1.000*
	Novel	c.*263_*264insA		3'UTR	0.5	N	*1.000*
	rs200675446	c.*263delA		3'UTR	4.9	N	*1.000*
	rs45564134	c.*974delG		3'UTR	0.5	N	*1.000*
	rs28465265	c.*274C>A		3'UTR	0.5	N	*1.000*
CYP2C19	rs3758581	c.991A>G	p.Ile331Val	missense	43.5	N	*<0.001*
	rs17884712	c.431G>A	p.Arg144His	missense	2.2	D	*1.000*
	rs576823729	c.648C>G	p.Cys216Trp	missense	0.5	N	*1.000*
	rs17882687	c.55A>C	p.Ile19Leu	missense	0.5	N	*1.000*
	rs17878459	c.276G>C	p.Glu92Asp	missense	3.3	N	*1.000*
	rs58973490	c.449G>A	p.Arg150His	missense	1.1	N	*1.000*
CYP2C8	rs1058932	c.*24C>T		3'UTR	23.9	N	*0.006*
	rs11572078	g.96827126dup		splicing	17.4	N	*<0.001*
	rs2071426	g.5932A>G		splicing	23.9	D	*1.000*
	rs10509681	c.890A>G	p.Lys297Arg	missense	4.9	N	*1.000*
	rs77147096	c.787G>A	p.Gly263Ser	missense	0.5	N	*1.000*
	rs369591911	c.65G>A	p.Arg22Gln	missense	0.5	N	*1.000*
	rs143386810	c.844G>A	p.Gly282Ser	missense	0.5	N	*1.000*
CYP2C9	rs1799853	c.430C>T	p.Arg144Cys	missense	8.8	D	*1.000*
	rs9332242	c.*108C>G		3'UTR	8.8	N	*1.000*
	rs28371685	c.1003C>T	p.Arg335Trp	missense	0.9	N	*1.000*
	rs1057910	c.1075A>C	p.Ile359Leu	missense	7.5	N	*0.475*
	rs577147873	c.*60C>T		3'UTR	0.4	N	*1.000*
	rs7900194	c.449G>A	p.Arg150His	missense	1.3	N	*1.000*
	rs2256871	c.752A>G	p.His251Arg	missense	2.2	D	*1.000*
	rs201055266	c.1034T>C	p.Met345Thr	missense	0.4	N	*1.000*
CYP2D6	rs16947	c.733C>T	p.Arg245Cys	missense	32.6	N	*0.211*
	rs769258	c.31G>A	p.Val11Met	missense	4.3	N	*0.119*
	rs1058172	c.941G>A	p.Arg314His	missense	4.9	D	*1.000*

Gene	rs code	NT change	AA change	Type	MAF (%)	*In silico* prediction	HWE p -value
	rs1065852	c.100C>T	p.Pro34Ser	missense	6.0	D	*0.224*
	Novel	c.551C>T		missense	0.5	N	*1.000*
	rs5030656	c.88_690del	p.Lys230_C442delins	nonframeshift deletion	1.1	LD	*1.000*
	rs28371717	c.556G>T	p.Ala186Ser	missense	0.5	N	*1.000*
	rs28371704	c.281A>G	p.His94Arg	missense	1.6	N	*1.000*
	rs3892097			splicing	2.2	D	*1.000*
	rs28371706	c.320C>T	p.Thr107Ile	missense	2.2	N	*1.000*
	rs139779104	c.482G>A	p.Gly161Glu	missense	0.5	N	*1.000*
	rs5030655	c.54del4>T	p.Trp152Gfs*2	frameshift deletion	0.5	D	*1.000*
	rs140513104	c.821C>T	p.Pro274Leu	missense	0.5	N	*1.000*
	rs59421388	c.859G>A	p.Val287Met	missense	0.5	N	*1.000*
	rs61736512	c.406G>A	p.Val136Met	missense	0.5	N	*1.000*
	rs28371703	c.271C>A	p.Leu91Met	missense	1.1	D	*1.000*
CYP3A4	rs28969391	c.*767delT		3'UTR	18.0	N	*0.757*
	rs28988604	c.*683C>T		3'UTR	3.5	N	*1.000*
	rs12721631	c.*329C>T		3'UTR	1.3	N	*1.000*
	rs4986907	c.485G>A	p.Arg162Gln	missense	0.4	N	*1.000*
	rs28371763	c.*948A>T		3'UTR	1.3	N	*1.000*
	rs28988606	c.*1095C>T		3'UTR	0.9	N	*1.000*
CYP3A5	rs15524	c.*14T>C		3'UTR	21.1	N	*0.576*
	rs776746	g.12083G>A		splicing	77.6	D	*0.431*
	rs41279857	c.299C>A	p.Ser100Tyr	missense	0.4	N	*1.000*
	rs10264272	g.19787G>A	p.Lys208Lys	synonym	3.1	N	*1.000*
	rs149664815	c.1378C>T	p.Gln460X	stop gain	0.4	D	*1.000*
	rs28371765	c.-3554A>C		5'UTR	0.4	N	*1.000*
	rs28365095	c.-3625G>A		5'UTR	0.4	N	*1.000*
	rs6977165	c.423A>G	p.X141Trp	stoploss	5.7	D	*1.000*
	rs145774441	c.827T>C	p.Ile276Thr	missense	0.4	N	*1.000*
	rs28371764	c.-3613C>T		5'UTR	3.1	N	*1.000*
	rs200579169	c.92dupG	p.Gly31fs	frameshift insertion	0.4	D	*1.000*
	rs28383468	c.88C>T	p.His30Tyr	missense	0.4	N	*1.000*
	rs147489136	c.608T>G	p.Phe203Cys	missense	0.4	N	*1.000*
	rs547253411	c.1372delG	p.Val458Sfs*16	frameshift deletion	0.4	D	*1.000*
	rs41303343	c.1035dupT	p.Thr346fs	frameshift insertion	1.8	D	*1.000*
	rs6957030	c.419T>G	p.Leu140Arg	missense	0.4	N	*1.000*
SLC15A1	rs1289389	c.*688G>A		3'UTR	19.0	N	*0.024*
	rs759932207	c.*178_*177delTT		3'UTR	19.6	N	*0.070*
	rs779338904	c.*178_*176delTTT		3'UTR	2.8	N	*1.000*
	Novel	c.*178_*179insT		3'UTR	1.1	N	*1.000*
	rs4646234	c.*598A>G		3'UTR	12.5	N	*1.000*
	rs2297322	c.350G>A	p.Ser117Asn	missense	18.5	N	*0.003*
	rs7331216	c.*59A>G		3'UTR	9.8	N	*0.517*
	rs113824127	c.*211G>T		3'UTR	1.1	N	*1.000*
	rs8187820	c.364G>A	p.Val122Met	missense	1.6	D	*1.000*

Gene	rs code	NT change	AA change	Type	MAF (%)	*In silico* prediction	HWE p -value
	rs8187838	c.1352C>A	p.Thr451Asn	missense	1.6	N	*1.000*
	Novel	c.*176_*177insT		3'UTR	3.8	N	*1.000*
	Novel	c.*174_*175insT		3'UTR	5.4	N	*1.000*
	Novel	c.*177delT		3'UTR	3.8	N	*1.000*
	Novel	c.*175delT		3'UTR	5.4	N	*1.000*
	Novel	c.*176_*175delTT		3'UTR	5.4	N	*1.000*
	Novel	c.*177_*175delTTT		3'UTR	5.4	N	*1.000*
	Novel	c.*178_*175delTTTT		3'UTR	5.4	N	*1.000*
	rs3783002	c.*224C>T		3'UTR	7.6	N	*0.346*
	rs4646227	c.1256G>C	p.Gly419Ala	missense	4.3	N	*1.000*
	rs2274828	c.1348G>A	p.Val450Ile	missense	0.5	N	*1.000*
	rs572627369	c.*160T>C		3'UTR	0.5	N	*1.000*
	rs578247729	c.*914C>T		3'UTR	0.5	N	*1.000*
	rs8187827	g.99354731T>C		splicing	0.5	N	*1.000*
	rs398037820	c.*178delT		3'UTR	3.9	N	*1.000*
	Novel	c.800A>T		missense	0.5	N	*1.000*
	rs114218227	c.*125G>A		3'UTR	1.1	N	*1.000*
	rs8187821	c.351C>A	p.Ser117Arg	missense	0.5	N	*1.000*
	Novel	c.*451G>A		3'UTR	0.5	N	*1.000*
	rs79136019	c.*587T>C		3'UTR	1.6	N	*1.000*
	rs8187815	c.-73T>C		5'UTR	1.1	N	*1.000*
	rs146304164	c.1246G>C	p.Val416Leu	missense	0.5	N	*1.000*
	Novel	c.*150_*144delCTTTTTC		3'UTR	0.5	N	*1.000*
	rs4646206	c.-33C>T		5'UTR	0.5	N	*1.000*
	Novel	c.*178_*173delTTTTTT		3'UTR	0.6	N	*1.000*
SLC22A1	rs628031	c.1222A>G	p.Met408Val	missense	66.8	N	*0.004*
	rs683369	c.480G>C	p.Leu160Phe	missense	85.9	N	*<0.001*
	rs776304541	c.1406G>A	p.Arg469His	missense	0.5	N	*1.000*
	rs35854239	c.1275_1276del	p.Pro425fs	splicing	45.7	D	*<0.001*
	rs34205214	c.1025G>A	p.Arg342His	missense	2.2	N	*1.000*
	rs34447885	c.41C>T	p.Ser14Phe	missense	2.2	N	*1.000*
	rs41267797	c.1390G>A	p.Val464Ile	missense	4.9	N	*0.151*
	rs72552763	c.1258_1260del	p.Met420del	nonframeshift deletion	18.5	D	*1.000*
	rs35270274	c.1463G>T	p.Arg488Met	missense	1.6	N	*1.000*
	rs35888596	c.113G>A	p.Gly38Asp	missense	2.2	D	*1.000*
	rs34059508	c.1393G>A	p.Gly465Arg	missense	1.1	D	*1.000*
	rs2282143	c.1022C>T	p.Pro341Leu	missense	1.1	D	*1.000*
	rs12208357	c.181C>T	p.Arg61Cys	missense	3.8	D	*0.090*
	rs36103319	c.659G>T	p.Gly220Val	missense	0.5	N	*1.000*
	rs78899680	c.1442G>T	p.Gly481Val	missense	0.5	N	*1.000*
	rs34130495	c.1201G>A	p.Gly401Ser	missense	0.5	N	*1.000*
	rs774654623	c.1396C>A	p.Pro466Thr	missense	0.5	N	*1.000*
SLC22A6	rs4149170	c.-127G>A		5'UTR	12.0	N	*0.009*
	rs4149171	c.-20A>G		5'UTR	16.8	N	*0.006*
	rs11568627	c.311C>T	p.Pro104Leu	missense	0.5	N	*1.000*

Gene	rs code	NT change	AA change	Type	MAF (%)	*In silico* prediction	HWE p -value
	rs150811286	c.*46T>C		3'UTR	0.5	N	*1.000*
	rs11568626	c.149G>A	p.Arg50His	missense	0.5	N	*1.000*
	rs181212822	c.*57G>A		3'UTR	0.5	N	*1.000*
SLC22A8	rs145493231	c.-857A>G		5'UTR	0.5	N	*1.000*
	Novel	c.*353C>T		3'UTR	0.5	N	*1.000*
	rs11568481	c.560C>T	p.Ala187Val	missense	0.5	N	*1.000*
	rs4149179	c.-16G>A		5'UTR	3.8	N	*0.090*
	rs45438191	c.473T>C	p.Val158Ala	missense	0.5	N	*1.000*
SLCO1B1	rs2306283	c.388A>G	p.Asn130Asp	missense	47.4	N	*0.354*
	rs4149056	c.521T>C	p.Val174Ala	missense	11.0	D	*0.355*
	rs4149087	c.*439T>G		3'UTR	38.2	N	*0.691*
	rs4149088	c.*463A>G		3'UTR	35.5	N	*1.000*
	rs11045819	c.463C>A	p.Pro155Thr	missense	13.2	N	*1.000*
	rs11045891	c.*449A>C		3'UTR	15.4	N	*0.725*
	rs11045852	c.733A>G	p.Ile245Val	missense	0.9	N	*1.000*
	rs74064213	c.1495A>G	p.Ile499Val	missense	0.9	N	*1.000*
	rs34671512	c.1929A>C	p.Leu643Phe	missense	5.3	N	*0.263*
	rs59502379	c.1463G>C	p.Gly488Ala	missense	1.8	D	*1.000*
	rs71581985	c.*46T>G		3'UTR	0.9	N	*1.000*
	rs77271279	g.21329832G>T		splicing	0.9	D	*1.000*
	rs61760249	c.*575G>A		3'UTR	0.4	N	*1.000*
	rs79135870	c.664A>G	p.Ile222Val	missense	0.4	N	*1.000*
	rs59113707	c.1200C>G	p.Phe400Leu	missense	0.4	N	*1.000*
	rs72655363	c.*82C>T		3'UTR	0.4	N	*1.000*
SLCO1B3	rs3764009	g.21013948C>T		splicing	16.3	N	*<0.001*
	rs4149117	c.250T>G	p.Ser84Ala	missense	76.1	N	*<0.001*
	rs4149158	c.-7_-4del-		5'UTR	24.5	N	*0.040*
	rs527574443	c.-28_-11del-		5'UTR	24.5	N	*0.040*
	rs7305323	c.-2125C>T		5'UTR	64.1	N	*<0.001*
	rs7311358	c.615G>A	p.Met205Ile	missense	72.8	N	*<0.001*
	rs397689574	c.*347_*348insA		3'UTR	32.6	N	*0.629*
	rs57585902	c.355A>G	p.Thr119Ala	missense	1.1	N	*1.000*
	rs60140950	c.767G>C	p.Gly228Ala	missense	14.7	D	*0.048*
	rs780598056	c.1333delG	p.Val445Sfs*6	frameshift deletion	0.5	D	*1.000*
	rs773176181	c.1247G>C	p.Gly416Ala	missense	0.5	N	*1.000*
	rs150007972	c.233C>A	p.Thr78Asn	missense	0.5	N	*1.000*
	rs61736817	c.1282C>T	p.Leu428Phe	missense	0.5	N	*1.000*
	rs76963574	c.1628C>G	p.Ala543Gly	missense	0.5	N	*1.000*
	rs115227445	c.592C>G	p.Leu198Val	missense	0.5	N	*1.000*
	rs77957556	c.*642G>A		3'UTR	1.1	N	*1.000*
	rs558592800	c.119_120insAATTG	p.Asp42Efs*12	frameshift insertion	0.5	D	*1.000*
	Novel	c.596G>T		missense	0.5	N	*1.000*
	Novel	c.-2107A>T		5'UTR	0.5	N	*1.000*
	rs12299012	c.1595T>C	p.Val532Ala	missense	1.1	N	*1.000*
	rs958332597	g.21032366C>T		splicing	0.5	N	*1.000*
SLCO2B1	rs11236359	c.-2866A>G		5'UTR	75.5	N	*<0.001*

Gene	rs code	NT change	AA change	Type	MAF (%)	*In silico* prediction	HWE p -value
	rs1944612	c.-36A>G		5'UTR	98.9	N	*<0.001*
	rs2851069	c.-71T>C		5'UTR	47.3	N	*0.078*
	rs17133818	c.*1386C>T		3'UTR	6.0	N	*1.000*
	rs1801906	c.*1070T>C		3'UTR	9.2	N	*1.000*
	rs2306168	c.1025C>T	p.Ser342Phe	missense	6.5	N	*0.263*
	rs3781727	c.*396T>C		3'UTR	6.5	N	*1.000*
	rs41298121	c.*1222T>C		3'UTR	10.3	N	*0.558*
	rs12422149	c.503G>A	p.Arg168Gln	missense	12.0	N	*0.595*
	Novel	c.*956C>A		3'UTR	0.5	N	*1.000*
	rs41298117	c.*721C>G		3'UTR	3.8	N	*1.000*
	rs78825186	c.485G>A	p.Arg162His	missense	1.1	N	*1.000*
	rs145875125	c.1206C>A	p.Asn402Lys	missense	0.5	N	*1.000*
	rs185838153	c.*1776T>C		3'UTR	0.5	N	*1.000*
	rs60113013	c._614del	p.Glu4_T6del	nonframeshift deletion	1.6	LD	*1.000*
	rs35199625	c.169G>A	p.Val57Met	missense	1.1	N	*1.000*
UGT1A3	rs6431625	c.140T>C	p.Val47Ala	missense	41.8	N	*0.212*
	rs28898619	c.342G>A	p.Met114Ile	missense	1.1	N	*1.000*
	rs3821242	c.31T>C	p.Trp11Arg	missense	45.7	N	*0.027*
	rs61764030	c.473C>T	p.Ala158Val	missense	1.1	N	*1.000*
	rs149324549	c.775G>C	p.Gly259Arg	missense	1.1	N	*1.000*
	rs45449995	c.808A>G	p.Met270Val	missense	2.2	D	*0.026*
	rs61764031	c.523A>T	p.Asn175Tyr	missense	0.5	N	*1.000*
	rs140541315	c.172G>A	p.Ala58Thr	missense	0.5	N	*1.000*
	rs13406898	c.431C>T	p.Thr144Ile	missense	0.5	N	*1.000*
	rs45595237	c.145C>T	p.Arg49Trp	missense	0.5	N	*1.000*
UGT2B7	rs57075995	c.*100_*101insA		3'UTR	17.9	N	*0.122*
	rs7439366	c.802T>C	p.Tyr268His	missense	62.0	N	*<0.001*
	Novel	c.*101delA		3'UTR	29.1	N	*<0.001*
	rs111878373	c.-2G>A		5'UTR	1.1	N	*1.000*
	rs140153012	c.321A>T	p.Leu107Phe	missense	1.1	N	*1.000*
	rs57075995	c.*100_*101insAA		3'UTR	2.2	N	*1.000*
	Novel	c.*101_*102insA		3'UTR	22.8	N	*0.012*
	Novel	c.*101_*102insAA		3'UTR	22.8	N	*0.012*
	rs60103519	c.536C>T	p.Thr179Ile	missense	1.1	N	*1.000*
	rs78265585	c.*247C>A		3'UTR	1.6	N	*1.000*

In silico functionality prediction was performed either using the functionality prediction score (FPS) for missense variants or dbNSFP v4.2 *in silico* algorithm for splice variants. Frameshift variants were considered deleterious. Nonframeshift variants were considered potentially deleterious. AA: amino acid; NT nucleotide; D: deleterious; HWE: Hardy-Weinberg equilibrium; LD: likely deleterious;/ MAF: minor allele frequency; N: neutral; PK: pharmacokinetics; UTR: untranslated region.

Table S9 FPS score of variants in PK-related genes identified in FH patients (n = 114).

Gene	rs code	NT change	AA change	Type	MAF (%)	FPS
ABCB1	rs2032582	c.2677T>G	p.Ser893Ala	missense	59.2	0.2
	rs2229107	c.3421T>A	p.Ser1141Thr	missense	1.3	0.0
	rs9282564	c.61A>G	p.Asn21Asp	missense	3.9	0.0
	rs2032582	c.2677T>A	p.Ser893Thr	missense	3.1	0.4
	rs2229109	c.1199G>A	p.Ser400Asn	missense	3.5	0.2
	rs35023033	c.2005C>T	p.Arg669Cys	missense	0.4	0.6
	rs35730308	c.3322T>C	p.Trp1108Arg	missense	0.4	0.8
	rs28364274	c.3751G>A	p.Val1251Ile	missense	0.9	0.2
	rs57521326	c.3262G>A	p.Asp1088Asn	missense	0.9	0.6
	rs36008564	c.781A>G	p.Ile261Val	missense	0.4	0.2
ABCB11	rs2287622	c.1331T>C	p.Val444Ala	missense	58.7	0.0
	rs11568364	c.2029A>G	p.Met677Val	missense	5.4	0.2
	rs1521808	c.3556G>A	p.Glu1186Lys	missense	0.5	0.4
	rs766285158	c.3691C>T	p.Arg1231Trp	missense	0.5	1.0
	rs11568357	c.616A>G	p.Ile206Val	missense	0.5	0.0
	rs111482608	c.1636C>A	p.Gln546Lys	missense	0.5	0.2
	rs11568370	c.1774G>C	p.Glu592Gln	missense	0.5	0.6
ABCC1	rs4148356	c.2168G>A	p.Arg723Gln	missense	0.5	0.0
	rs45511401	c.2012G>T	p.Gly671Val	missense	3.8	0.8
	rs183032276	c.4154G>A	p.Arg1385Gln	missense	0.5	1.0
	rs112282109	c.1898G>A	p.Arg633Gln	missense	0.5	0.0
	rs13337489	c.3140G>C	p.Cys1047Ser	missense	1.1	0.0
	rs28706727	c.3436G>A	p.Val1146Ile	missense	0.5	0.4
	Novel	c.145T>G	p.Cys49Gly	missense	0.5	NR
	rs187769078	c.185G>A	p.Arg62Gln	missense	0.5	0.4
	rs199815778	c.4441G>A	p.Val1481Ile	missense	0.5	0.4
ABCC2	rs2273697	c.1249G>A	p.Val417Ile	missense	16.8	0.0
	rs45441199	c.3107T>C	p.Ile1036Thr	missense	1.1	0.2
	rs927344	c.116A>T	p.Tyr39Phe	missense	98.9	0.2
	rs17222723	c.3563T>A	p.Val1188Glu	missense	7.6	0.2
	rs8187699	c.3817A>G	p.Thr1273Ala	missense	0.5	0.2
	rs8187710	c.4544G>A	p.Cys1515Tyr	missense	9.8	0.2
	rs17222617	c.2546T>G	p.Leu849Arg	missense	1.6	0.4
	rs8187692	c.3542G>T	p.Arg1181Leu	missense	2.7	0.8
	rs7080681	c.1058G>A	p.Arg353His	missense	2.7	0.0
	rs17216317	c.3872C>T	p.Pro1291Leu	missense	3.3	0.8
	rs72558199	c.3196C>T	p.Arg1066X	stop gain	0.5	0.5
	rs141413284	c.1860T>A	p.Asp620Glu	missense	0.5	0.2
ABCC3	rs34926034	c.202C>T	p.His68Tyr	missense	1.1	0.0
	rs141856639	c.3971G>A	p.Arg1324His	missense	1.1	1.0
	rs35999272	c.2758C>T	p.Pro920Ser	missense	2.2	0.0
	rs34346931	c.1223A>G	p.Glu408Gly	missense	0.5	1.0
	rs150601692	c.4030A>G	p.Lys1344Glu	missense	0.5	0.0
	rs11568591	c.3890G>A	p.Arg1297His	missense	6.5	0.8
	rs200779271	c.980T>C	p.Ile327Thr	missense	0.5	0.0
	rs201562834	c.871C>T	p.Arg291Trp	missense	0.5	0.2
	rs1003354	c.1580C>T	p.Thr527Met	missense	0.5	0.4
	rs143608762	c.694C>T	p.Arg232Trp	missense	0.5	0.8
	rs35777968	c.296G>A	p.Arg99Gln	missense	0.5	0.0
	rs139106724	c.2377G>A	p.Val793Ile	missense	1.1	0.4
	rs200413276	c.2558C>A	p.Ala853Asp	missense	0.5	0.6
	rs372683132	c.922G>A	p.Gly308Ser	missense	1.1	0.4
	rs11568584	c.2153A>T	p.Lys718Met	missense	0.5	0.4
	rs11568590	c.4094A>G	p.Gln1365Arg	missense	0.5	0.0
	rs11568608	c.1820G>A	p.Ser607Asn	missense	1.1	0.0
	rs34291385	c.2293G>C	p.Val765Leu	missense	1.1	0.4
	rs200903266	c.3401G>A	p.Arg1134Gln	missense	0.5	1.0
	rs11568588	c.4042C>T	p.Arg1348Cys	missense	1.1	0.2

Gene	rs code	NT change	AA change	Type	MAF (%)	FPS
	rs148804178	c.205C>G	p.Leu69Val	missense	0.5	0.6
ABCG2	rs45605536	c.1582G>A	p.Ala528Thr	missense	1.1	0.4
	rs2231142	c.421C>A	p.Gln141Lys	missense	6.5	0.2
	rs2231137	c.34G>A	p.Val12Met	missense	6	0.2
	rs1337337886	c.131A>G	p.Tyr44Cys	missense	0.5	0.8
	rs35965584	c.1624A>G	p.Thr542Ala	missense	0.5	0.4
	Novel	c.1453C>A	p.Pro485Thr	missense	0.5	NR
	rs138606116	c.1060G>A	p.Gly354Arg	missense	0.5	0.0
	rs34783571	c.1858G>A	p.Asp620Asn	missense	0.5	0.4
	rs34264773	c.1758A>T	p.Lys586Asn	missense	0.5	0.3
CYP1A2	rs17861157	c.894C>A	p.Ser298Arg	missense	3.3	0.2
	rs45540640	c.613T>G	p.Phe205Val	missense	0.5	1.0
	rs201763966	c.142T>G	p.Trp48Gly	missense	0.5	0.8
	rs758124536	c.409C>T	p.Arg137Trp	missense	0.5	1.0
CYP2C19	rs3758581	c.991G>G	p.Val331Val	missense	43.5	NR
	rs17884712	c.431G>A	p.Arg144His	missense	2.2	0.8
	rs576823729	c.648C>G	p.Cys216Trp	missense	0.5	0.6
	rs17882687	c.55A>C	p.Ile19Leu	missense	0.5	0.0
	rs17878459	c.276G>C	p.Glu92Asp	missense	3.3	0.2
	rs58973490	c.449G>A	p.Arg150His	missense	1.1	0.0
CYP2C8	rs11572103	c.499A>T	p.Ile167Phe	missense	3.3	0.4
	rs10509681	c.890A>G	p.Lys297Arg	missense	4.9	0.2
	rs11572080	c.110G>A	p.Arg37Lys	missense	5.4	0.4
	rs77147096	c.787G>A	p.Gly263Ser	missense	0.5	0.0
	rs1058930	c.486C>G	p.Ile162Met	missense	4.9	0.6
	rs369591911	c.65G>A	p.Arg22Gln	missense	0.5	1.0
	rs143386810	c.844G>A	p.Gly282Ser	missense	0.5	0.8
CYP2C9	rs1799853	c.430C>T	p.Arg144Cys	missense	8.8	1.0
	rs28371685	c.1003C>T	p.Arg335Trp	missense	0.9	0.6
	rs1057910	c.1075A>C	p.Ile359Leu	missense	7.5	0.2
	rs7900194	c.449G>A	p.Arg150His	missense	1.3	0.0
	rs2256871	c.752A>G	p.His251Arg	missense	2.2	0.8
	rs201055266	c.1034T>C	p.Met345Thr	missense	0.4	1.0
	rs28371686	c.1080C>G	p.Asp360Glu	missense	0.4	0.8
	rs9332239	c.1465C>T	p.Pro489Ser	missense	0.4	0.8
CYP2D6	rs16947	c.733T>T	p.Cys245Cys	missense	32.6	NR
	rs769258	c.31G>A	p.Val11Met	missense	4.3	0.0
	rs1058172	c.941G>A	p.Arg314His	missense	4.9	1.0
	rs1065852	c.100C>T	p.Pro34Ser	missense	6	1.0
	Novel	c.551C>T	p.Ala184Val	missense	0.5	
	rs28371717	c.556G>T	p.Ala186Ser	missense	0.5	0.0
	rs28371704	c.281A>G	p.His94Arg	missense	1.6	0.0
	rs28371706	c.320C>T	p.Thr107Ile	missense	2.2	0.0
	rs139779104	c.482G>A	p.Gly161Glu	missense	0.5	0.6
	rs140513104	c.821C>T	p.Pro274Leu	missense	0.5	1.0
	rs59421388	c.859G>A	p.Val287Met	missense	0.5	0.4
	rs61736512	c.406G>A	p.Val136Ile	missense	0.5	0.0
	rs28371703	c.271C>A	p.Leu91Met	missense	1.1	0.6
CYP3A4	rs4986907	c.485G>A	p.Arg162Gln	missense	0.4	0.0
CYP3A5	rs41279857	c.299C>A	p.Ser100Tyr	missense	0.4	0.8
	rs149664815	c.1378C>T	p.Gln460X	stop gain	0.4	1.0
	rs6977165	c.423A>G	p.X141Trp	stoploss	5.7	1.0
	rs145774441	c.827T>C	p.Ile276Thr	missense	0.4	0.6
	rs28383468	c.88C>T	p.His30Tyr	missense	0.4	0.0
	rs147489136	c.608T>G	p.Phe203Cys	missense	0.4	1.0
	rs6957030	c.419T>G	p.Leu140Arg	missense	0.4	0.0
SLC15A1	rs2297322	c.350G>A	p.Ser117Asn	missense	18.5	0.0
	rs8187820	c.364G>A	p.Val122Met	missense	1.6	0.6
	rs8187838	c.1352C>A	p.Thr451Asn	missense	1.6	0.0

Gene	rs code	NT change	AA change	Type	MAF (%)	FPS
	rs4646227	c.1256G>C	p.Gly419Ala	missense	4.3	0.0
	rs2274828	c.1348G>A	p.Val450Ile	missense	0.5	0.0
	Novel	c.800A>T	p.Glu267Val	missense	0.5	NR
	rs8187821	c.351C>A	p.Ser117Arg	missense	0.5	0.0
	rs146304164	c.1246G>C	p.Val416Leu	missense	0.5	0.0
SLC22A1	rs628031	c.1222A>G	p.Met408Val	missense	66.8	0.0
	rs683369	c.480G>C	p.Leu160Phe	missense	85.9	0.0
	rs776304541	c.1406G>A	p.Arg469His	missense	0.5	0.5
	rs34205214	c.1025G>A	p.Arg342His	missense	2.2	0.0
	rs34447885	c.41C>T	p.Ser14Phe	missense	2.2	0.2
	rs41267797	c.1390G>A	p.Val464Ile	missense	4.9	0.0
	rs35270274	c.1463G>T	p.Arg488Met	missense	1.6	0.0
	rs35888596	c.113G>A	p.Gly38Asp	missense	2.2	1.0
	rs34059508	c.1393G>A	p.Gly465Arg	missense	1.1	0.8
	rs2282143	c.1022C>T	p.Pro341Leu	missense	1.1	0.8
	rs12208357	c.181C>T	p.Arg61Cys	missense	3.8	0.6
	rs36103319	c.659G>T	p.Gly220Val	missense	0.5	0.8
	rs78899680	c.1442G>T	p.Gly481Val	missense	0.5	0.3
	rs34130495	c.1201G>A	p.Gly401Ser	missense	0.5	0.8
	rs774654623	c.1396C>A	p.Pro466Thr	missense	0.5	0.0
SLC22A6	rs11568627	c.311C>T	p.Pro104Leu	missense	0.5	0.6
	rs11568626	c.149G>A	p.Arg50His	missense	0.5	0.6
SLC22A8	rs11568481	c.560C>T	p.Ala187Val	missense	0.5	0.0
	rs45438191	c.473T>C	p.Val158Ala	missense	0.5	0.0
SLCO1B1	rs2306283	c.388A>G	p.Asn130Asp	missense	47.4	0.0
	rs4149056	c.521T>C	p.Val174Ala	missense	11	0.8
	rs11045819	c.463C>A	p.Pro155Thr	missense	13.2	0.2
	rs11045852	c.733A>G	p.Ile245Val	missense	0.9	0.2
	rs74064213	c.1495A>G	p.Ile499Val	missense	0.9	0.0
	rs34671512	c.1929A>C	p.Leu643Phe	missense	5.3	0.0
	rs59502379	c.1463G>C	p.Gly488Ala	missense	1.8	0.8
	rs79135870	c.664A>G	p.Ile222Val	missense	0.4	0.0
	rs59113707	c.1200C>G	p.Phe400Leu	missense	0.4	0.0
SLCO1B3	rs4149117	c.250T>G	p.Ser84Ala	missense	76.1	0.2
	rs7311358	c.615G>A	p.Met205Ile	missense	72.8	0.0
	rs57585902	c.355A>G	p.Thr119Ala	missense	1.1	0.0
	rs60140950	c.767G>C	p.Gly228Ala	missense	14.7	1.0
	rs773176181	c.1247G>C	p.Gly416Ala	missense	0.5	0.8
	rs150007972	c.233C>A	p.Thr78Asn	missense	0.5	0.4
	rs61736817	c.1282C>T	p.Leu428Phe	missense	0.5	0.0
	rs76963574	c.1628C>G	p.Ala543Gly	missense	0.5	0.8
	rs115227445	c.592C>G	p.Leu198Val	missense	0.5	0.0
	Novel	c.596G>T	p.Gly199Val	missense	0.5	NR
	rs12299012	c.1595T>C	p.Val532Ala	missense	1.1	0.0
SLCO2B1	rs2306168	c.1025C>T	p.Ser342Phe	missense	6.5	0.0
	rs12422149	c.503G>A	p.Arg168Gln	missense	12	0.2
	rs78825186	c.485G>A	p.Arg162His	missense	1.1	0.0
	rs145875125	c.1206C>A	p.Asn402Lys	missense	0.5	0.0
	rs35199625	c.169G>A	p.Val57Met	missense	1.1	0.4
UGT1A3	rs6431625	c.140T>C	p.Val47Ala	missense	41.8	0.0
	rs28898619	c.342G>A	p.Met114Ile	missense	1.1	0.0
	rs3821242	c.31T>C	p.Trp11Arg	missense	45.7	0.0
	rs61764030	c.473C>T	p.Ala158Val	missense	1.1	0.3
	rs149324549	c.775G>C	p.Gly259Arg	missense	1.1	0.3
	rs45449995	c.808A>G	p.Met270Val	missense	2.2	0.8
	rs61764031	c.523A>T	p.Asn175Tyr	missense	0.5	0.0
	rs140541315	c.172G>A	p.Ala58Thr	missense	0.5	0.0
	rs13406898	c.431C>T	p.Thr144Ile	missense	0.5	0.3
	rs45595237	c.145C>T	p.Arg49Trp	missense	0.5	0.5

Gene	rs code	NT change	AA change	Type	MAF (%)	FPS
UGT2B7	rs7439366	c.802T>C	p.Tyr268His	missense	62	0.3
	rs140153012	c.321A>T	p.Leu107Phe	missense	1.1	0.0
	rs60103519	c.536C>T	p.Thr179Ile	missense	1.1	0.0

AA: amino acid; NT nucleotide; NR: not reported (for variants that did not show any prediction in the 5 algorithms used); FPS: functionality prediction score; MAF: minor allele frequency; PK: pharmacokinetics.

Table S10 Influence of deleterious variants in PK-related genes on LDL-c reduction in FH patients on statin treatment.

Gene	rs code	NT change	Type	LDL-c reduction (%)			*Adjusted*	Prediction
				Non carriers	Carriers	*p-value*	*p-value*	
**All statins**								
ABCC1	rs45511401	c.2012G>T	missense	-45.9 ± 20.1 (85)	-64.7 ± 6.4 (7)	*<0.0001*	*0.001*	0.8
ABCC2	rs17216317	c.3872C>T	missense	-48.3 ± 18.9 (86)	-33.6 ± 30.7 (6)	*0.297*	*1.000*	0.8
	rs8187692	c.3542G>T	missense	-47.5 ± 19.9 (87)	-43 ± 23.5 (5)	*0.693*	*0.912*	0.8
ABCC3	rs11568591	c.3890G>A	missense	-47 ± 20.3 (80)	-49.5 ± 18.2 (12)	*0.665*	*0.950*	0.8
CYP2C19	rs17884712	c.431G>A	missense	-47.9 ± 19.8 (88)	-33.5 ± 22.2 (4)	*0.286*	*1.000*	0.8
CYP2C8	rs1058930	c.486C>G	missense	-47.7 ± 20.2 (84)	-43.4 ± 18.1 (8)	*0.548*	*1.000*	0.6
	rs2071426	g.96828323T>C	splicing	-47.7 ± 19.2 (52)	-46.7 ± 21.3 (40)	*0.816*	*0.906*	D
CYP2C9	rs1799853	c.430C>T	missense	-48.8 ± 19.3 (95)	-43.6 ± 19.6 (19)	*0.298*	*1.000*	1.0
	rs2256871	c.752A>G	missense	-47.7 ± 19.4 (109)	-53.4 ± 19.5 (5)	*0.558*	*0.962*	0.8
CYP2D6	rs3892097	g.42524947C>T	splicing	-47.8 ± 19.9 (88)	-36.5 ± 22.3 (4)	*0.387*	*1.000*	D
	rs1058172	c.941G>A	missense	-46.9 ± 19.8 (83)	-51.1 ± 22.9 (9)	*0.608*	*1.000*	1.0
	rs1065852	c.100C>T	missense	-47.2 ± 19.9 (82)	-48.3 ± 22 (10)	*0.875*	*0.931*	1.0
CYP3A5	rs776746	g.99270539C>T	splicing	-57.5 ± 16.7 (7)	-47.4 ± 19.4 (107)	*0.168*	*1.000*	D
	rs41303343	c.035dup	frameshift insertion	-47.8 ± 19.5 (110)	-51.9 ± 15 (4)	*0.634*	*0.990*	D
	rs6977165	c.423A>G	stoploss	-47.8 ± 19.5 (101)	-49.1 ± 18.7 (13)	*0.823*	*0.901*	1.0
SLC15A1	rs8187820	c.364G>A	missense	-47.3 ± 20.2 (89)	-47.1 ± 13.7 (3)	*0.983*	*1.000*	0.6
SLC22A1	rs35888596	c.113G>A	missense	-46.8 ± 20.1 (88)	-59.2 ± 12.9 (4)	*0.147*	*1.000*	1.0
	rs72552763	c.258_1260del	non-frameshift deletion	-48.4 ± 20.2 (60)	-45.3 ± 19.8 (32)	*0.484*	*1.000*	D
	rs12208357	c.181C>T	missense	-47.5 ± 19.9 (86)	-44.5 ± 23 (6)	*0.768*	*0.937*	0.6
SLCO1B1	rs4149056	c.521T>C	missense	-47.5 ± 19.6 (89)	-49.6 ± 18.7 (25)	*0.633*	*1.000*	0.8
	rs59502379	c.1463G>C	missense	-48.1 ± 19.4 (110)	-45.4 ± 20.3 (4)	*0.808*	*0.940*	0.8
SLCO1B3	rs60140950	c.683G767G>C	missense	-46.3 ± 19.3 (69)	-50.4 ± 22.2 (23)	*0.432*	*1.000*	1.0
UGT1A3	rs45449995	c.808A>G	missense	-47.2 ± 20.2 (89)	-50.1 ± 13.5 (3)	*0.753*	*0.942*	0.8
**Atorvastatin**								
ABCC1	rs45511401	c.2012G>T	missense	-46.3 ± 18.4 (72)	-65.8 ± 6.2 (6)	*0.000*	*0.001*	0.8
ABCC2	rs8187692	c.3542G>T	missense	-48.4 ± 18.1 (73)	-39.6 ± 24.1 (5)	*0.467*	*1.000*	0.8
	rs17216317	c.3872C>T	missense	-48.2 ± 18.3 (73)	-42.7 ± 23.4 (5)	*0.637*	*0.965*	0.8
ABCC3	rs11568591	c.3890G>A	missense	-47.8 ± 18.3 (69)	-47.9 ± 21.3 (9)	*0.988*	*0.988*	0.8
CYP2C19	rs17884712	c.431G>A	missense	-48.6 ± 18.1 (74)	-33.5 ± 22.2 (4)	*0.269*	*1.000*	0.8
CYP2C8	rs2071426	g.96828323T>C	splicing	-46.5 ± 17 (43)	-49.4 ± 20.4 (35)	*0.512*	*1.000*	D
	rs1058930	c.486C>G	missense	-48.1 ± 18.6 (71)	-44.7 ± 19.2 (7)	*0.665*	*0.924*	0.6
CYP2C9	rs1799853	c.430C>T	missense	-48.9 ± 17.8 (81)	-42.8 ± 20.5 (17)	*0.264*	*1.000*	1.0
	rs1799853	c.430C>T	missense	-48.9 ± 17.8 (81)	-42.8 ± 20.5 (17)	*0.264*	*1.000*	1.0
	rs2256871	c.752A>G	missense	-47.6 ± 18.3 (93)	-53.4 ± 19.5 (5)	*0.547*	*1.000*	0.8
CYP2D6	rs3892097	g.42524947C>T	splicing	-48.4 ± 18.3 (74)	-36.5 ± 22.3 (4)	*0.364*	*1.000*	D
	rs1065852	c.100C>T	missense	-48.4 ± 18.7 (69)	-43.6 ± 17.1 (9)	*0.452*	*1.000*	1.0
	rs1058172	c.941G>A	missense	-48.2 ± 18.6 (71)	-43.7 ± 18.5 (7)	*0.553*	*0.988*	1.0

**Gene**	**rs code**	**NT change**	**Type**	**LDL-c reduction (%)**				
				**Non carriers**	**Carriers**	**p-*value***	***Adjusted p-value***	**Prediction**
CYP3A5	rs776746	NA	splicing	-57.5 ± 16.7 (7)	-47.1 ± 18.3 (91)	*0.160*	*1.000*	D
	rs41303343	c.035dup	frameshift insertion	-47.7 ± 18.3 (95)	-52 ± 20.4 (3)	*0.751*	*0.963*	D
SLC22A1	rs35888596	c.113G>A	missense	-47.2 ± 18.6 (74)	-59.2 ± 12.9 (4)	*0.158*	*1.000*	1.0
	rs12208357	c.181C>T	missense	-47.2 ± 18.5 (75)	-62.3 ± 15.4 (3)	*0.228*	*1.000*	0.6
	rs72552763	c.258_1260de1>l	nonframeshift deletion	-48.5 ± 19.2 (51)	-46.5 ± 17.4 (27)	*0.649*	*0.954*	D
	rs72552763	c.258_1260de1>l	nonframeshift deletion	-48.5 ± 19.2 (51)	-46.5 ± 17.4 (27)	*0.649*	*0.954*	D
SLCO1B1	rs4149056	c.521T>C	missense	-47.2 ± 18.5 (78)	-50.3 ± 17.7 (20)	*0.502*	*1.000*	0.8
	rs59502379	c.1463G>C	missense	-48 ± 18.3 (94)	-45.4 ± 20.3 (4)	*0.815*	*0.927*	0.8
SLCO1B3	rs60140950	c.683G767G>C	missense	-47 ± 17.7 (63)	-51.4 ± 21.8 (15)	*0.473*	*1.000*	1.0
ABCC3	rs11568591	c.3890G>A	missense	-35.8 ± 16.7 (22)	-33.5 ± 23.6 (3)	*0.884*	*0.921*	0.8
CYP2C8	rs2071426	g.96828323T>C	splicing	-36.3 ± 15.2 (15)	-34.3 ± 20.3 (10)	*0.790*	*0.941*	D
CYP2C9	rs1799853	c.430C>T	missense	-37.9 ± 20.2 (30)	-31.2 ± 18.3 (9)	*0.360*	*1.000*	1.0
CYP3A5	rs776746	g.99270539C>T	splicing	-33.8 ± 26 (3)	-36.6 ± 19.6 (36)	*0.870*	*0.946*	D
SLC22A1	rs72552763	c.1258_1260del	nonframeshift deletion	-37.4 ± 15.6 (19)	-29.5 ± 21.4 (6)	*0.432*	*1.000*	D
	rs12208357	c.181C>T	missense	-36.4 ± 17.5 (22)	-29.5 ± 14.2 (3)	*0.503*	*1.000*	0.6
SLCO1B1	rs4149056	c.521T>C	missense	-35.4 ± 19.2 (27)	-38.6 ± 21.6 (12)	*0.668*	*0.903*	0.8
SLCO1B3	rs60140950	c.683G767G>C	missense	-37.1 ± 18.4 (20)	-29.3 ± 8.3 (5)	*0.178*	*1.000*	1.0

FH patients carrying the homozygous form of the minor allele (AA) were grouped with the heterozygous carriers (RA) and compared with non-carriers (RR). Continuous variables are shown as mean ± SD and were compared by *t*-test. The p-value was adjusted using the Benjamini-Hochberg correction. *In silico* functionality prediction was performed either using the functionality prediction score (FPS) for missense variants or dbNSFP v4.2 *in silico* algorithm for splice variants. Frameshift variants were considered deleterious. Nonframeshift variants were considered potentially deleterious. NT nucleotide; D: deleterious; FPS: Functionality prediction score; N: neutral.

Table S11 Influence of variants in PK-related genes in genetic and non-genetic variables on LDL-c reduction in FH patients: Univariate linear regression analysis.

**Variant**		**β**	**SE**	***p-value***	***Adjusted p-value***
**Deleterious variants**					
*CYP2C19*9* c.431G>A	A allele	14.4	10.2	*0.159*	*0.520*
CYP2C8 c.486C>G	A allele	4.2	7.4	*0.570*	*0.855*
CYP2C8 g.5932A>G	G allele	1	4.2	*0.813*	*0.915*
CYP2C9 c.430C>T	T allele	4.2	7.4	*0.570*	*0.606*
CYP2C9 c.752A>G	G allele	5.2	4.9	*0.286*	*0.859*
CYP2D6 c.941G>A	A allele	-5.7	8.9	*0.525*	*0.862*
CYP2D6 c.100C>T	T allele	-4.2	7.0	*0.551*	*0.940*
CYP2D6 g.6866G>A	A allele	11.3	10.2	*0.270*	*0.608*
CYP3A5 c.624G>A	A allele	-1.2	6.7	*0.862*	*0.912*
CYP3A5 c.423A>G	G allele	-1.3	5.7	*0.827*	*0.924*
*CYP3A5*3* g.12083G>A	A allele	4.6	7.6	*0.684*	*0.504*
UGT1A3 c.808A>G	G allele	-2.9	11.8	*0.182*	*0.504*
ABCC1 c.2012G>T	T allele	-18.8	7.7	***0.016***	***0.096***
ABCC2 c.3872C>T	T allele	14.7	8.3	*0.082*	*0.328*
ABCC2 c.3542G>T	T allele	4.5	9.2	*0.625*	*0.900*
ABCC3 c.3890G>A	A allele	-2.5	6.2	*0.685*	*0.881*
SLC15A1 c.364G>A	A allele	0.2	11.8	*0.987*	*0.987*
SLC22A1 c.181C>T	T allele	3	8.5	*0.726*	*0.901*
SLC22A1 c.113G>A	A allele	-12.5	10.2	*0.224*	*0.538*
SLC22A1 c.1260_1262del	Deletion	3.1	4.4	*0.485*	*0.831*
*SLCO1B1*5* c.521T>C	C allele	-2.1	4.4	*0.641*	*0.888*
SLCO1B1 c.1463G>C	C allele	2.7	9.9	*0.784*	*0.941*
SLCO1B3 c.767G>C	C allele	-4.1	4.8	*0.396*	*0.750*
**Treatment**					
Baseline LDL-c		-0.1	0.02	***<0.001***	***<0.001***
High intensity treatment		-15.8	5.0	***0.002***	***0.024***
Atorvastatin		-7.3	5.9	*0.218*	*0.561*
Rosuvastatin		-16.4	8.0	***0.043***	*0.193*
Ezetimibe		-8.8	3.7	***0.018***	***0.090***
Drug interactions	CYP3A4 inhibitor	-8.9	6.4	*0.164*	*0.590*
SRAE	Presence	-11.8	4.3	***0.007***	***0.063***
	Myopathy	-11.8	4.7	*0.014*	*0.101*
Reduced adherence		14.4	10.2	*0.159*	*0.919*
**Patient characteristics**					
Age		0.11	0.1	*0.391*	*0.834*
Gender	Male	-3.3	4.0	*0.413*	*0.834*
Ethnics	Brown	7.2	4.3	*0.099*	*0.317*
	Black	-0.4	5.6	*0.942*	*0.972*
Type 2 diabetes		-2.8	4.5	*0.525*	*0.859*
BMI		1.44	0.4	***<0.001***	***<0.001***
FH-related variant	Carrier	-4.2	3.9	*0.288*	*0.627*

β: linear coefficient; SE: standard error; BMI: body mass index; FH: familial hypercholesterolemia; LDL-c: low-density lipoprotein cholesterol; SRAE: statin-related adverse events. P-value was adjusted using the Benjamini-Hochberg correction.

Table S12 Influence of deleterious variants (MAF > 1.0%) on LDL-c response to statins in FH patients: Multivariate linear regression analysis.

**Variant**		**n**	**β**	**SE**	***p-value***
CYP2C19 c.431G>A	A allele	92	14.4	8.7	*0.101*
CYP2C8 c.486C>G	A allele	92	-2.2	6.5	*0.737*
CYP2C8 g.5932A>G	G allele	92	0.6	3.7	*0.863*
*CYP2C9*2* c.430C>T	T allele	114	2.3	4.2	*0.595*
*CYP2C9*9* c.752A>G	G allele	114	9	7.5	*0.232*
CYP2D6 c.941G>A	A allele	92	-6.5	6.0	*0.281*
CYP2D6 c.100C>T	T allele	92	-6.4	5.8	*0.272*
CYP2D6 g.6866G>A	A allele	92	0.3	90.	*0.974*
CYP3A5 c.624G>A	A allele	114	-1.3	8.1	*0.873*
CYP3A5 c.423A>G	G allele	114	-2.1	4.9	*0.669*
*CYP3A5*3* g.12083G>A	A allele	114	4.7	6.4	*0.463*
UGT1A3 c.808A>G	G allele	92	-11.3	10.3	*0.274*
ABCC1 c.2012G>T	T allele	92	-11.5	6.7	*0.092*
ABCC2 c.3872C>T	T allele	92	12.2	7.2	*0.095*
ABCC2 c.3542G>T	T allele	92	3.6	8.0	*0.656*
ABCC3 c.3890G>A	A allele	92	-2.0	5.3	*0.710*
SLC15A1 c.364G>A	A allele	92	-11.8	10.3	*0.253*
SLC22A1 c.181C>T	T allele	92	-2.3	7.3	*0.757*
SLC22A1 c.113G>A	A allele	92	-10.3	8.8	*0.247*
SLC22A1 c.1260_1262del	Deletion	92	-1.4	4.0	*0.720*
*SLCO1B1*5* c.521T>C	C allele	114	-3.3	3.7	*0.365*
SLCO1B1 c.1463G>C	C allele	114	3.1	8.1	*0.701*
SLCO1B3 c.767G>C	C allele	92	-6.3	4.3	*0.150*

Each model was adjusted with the following covariates: body mass index, baseline LDL-c, therapy intensity, and presence of SRAE. n: number of patients; β: linear coefficient; SE: standard error; LDL-c: low-density lipoprotein cholesterol; FH: familial hypercholesterolemia; SRAE: statin-related adverse events.

Table S13 Association of variants in PK-related genes and non-genetic variables with statin response in FH patients: Univariate logistic regression analysis.

**Variable**		**RE,** % **(58)**	**NRE,** % **(56)**	**OR (95%CI)**	***p-value***	***Adjusted p-value***
**Deleterious variants**						
*CYP2C19*9* c.431G>A	A allele	2.2 (1)	6.4 (3)	3.0 (0.4 - 61.9)	*0.349*	*0.785*
CYP2C8 c.486C>G	A allele	4.4 (2)	12.8 (6)	3.1 (0.7 - 22.3)	*0.175*	*0.700*
CYP2C8 g.5932A>G	G allele	44.4 (20)	42.6 (20)	0.9 (0.4 - 2.1)	*0.855*	*0.993*
CYP2C9 c.430C>T	T allele	12.1 (7)	21.4 (12)	2.0 (0.7 - 5.8)	*0.185*	*0.666*
CYP2C9 c.752A>G	G allele	5.2 (3)	3.6 (2)	0.7 (0.1 - 4.3)	*0.678*	*0.939*
CYP2D6 c.941G>A	A allele	11.1 (5)	8.5 (4)	0.7 (0.2 - 3)	*0.676*	*0.973*
CYP2D6 c.100C>T	T allele	11.1 (5)	10.6 (5)	1.0 (0.2 - 3.7)	*0.942*	*1.000*
CYP2D6 g.6866G>A	A allele	2.2 (1)	6.4 (3)	3.0 (0.4 - 61.9)	*0.349*	*0.739*
CYP3A5 c.624G>A	A allele	3.4 (2)	3.6 (2)	1.0 (0.1 - 8.9)	*0.972*	*1.000*
CYP3A5 c.423A>G	G allele	10.3 (6)	12.5 (7)	1.2 (0.4 - 4.1)	*0.718*	*0.909*
*CYP3A5*3* g.12083G>A	A allele	93.1 (54)	94.6 (53)	1.3 (0.3 - 6.9)	*0.733*	*0.880*
UGT1A3 c.808A>G	G allele	4.4 (2)	2.1 (1)	0.5 (0.0 - 5)	*0.541*	*0.885*
ABCC1 c.2012G>T	T allele	15.6 (7)	0.0 (0)	-	-	-
ABCC2 c.3872C>T	T allele	2.2 (1)	10.6 (5)	5.2 (0.8 - 102.6)	*0.138*	*0.475*
ABCC2 c.3542G>T	T allele	4.4 (2)	6.4 (3)	1.5 (0.2 - 11.5)	*0.683*	*0.921*
ABCC3 c.3890G>A	A allele	13.3 (6)	12.8 (6)	1.0 (0.3 - 3.3)	*0.936*	*1.000*
SLC15A1 c.364G>A	A allele	4.4 (2)	2.1 (1)	0.5 (0 - 5)	*0.541*	*0.927*
SLC22A1 c.181C>T	T allele	4.4 (2)	8.5 (4)	2 (0.4 - 15)	*0.437*	*0.874*
SLC22A1 c.113G>A	A allele	6.7 (3)	2.1 (1)	0.3 (0 - 2.5)	*0.311*	*0.746*
SLC22A1 c.1260_1262del	Deletion	31.1 (14)	38.3 (18)	1.4 (0.6 - 3.3)	*0.470*	*0.891*
*SLCO1B1*5* c.521T>C	C allele	24.1 (14)	19.6 (11)	0.8 (0.3 - 1.9)	*0.563*	*0.844*
SLCO1B1 c.1463G>C	C allele	3.4 (2)	3.6 (2)	1 (0.1 - 8.9)	*0.972*	*1.000*
SLCO1B3 c.767G>C	C allele	26.7 (12)	23.4 (11)	0.8 (0.3 - 2.2)	*0.718*	*0.891*
**Treatment**						
Baseline LDL-c (mg/dL)		275 ± 90	226 ± 61	0.99 (0.98 – 0.99)	***0.002***	***0.024***
High intensity treatment		93.1 (54)	78.6 (44)	0.3 (0.1 - 0.8)	***0.033***	*0.198*
Atorvastatin		77.6 (45)	82.1 (46)	1.3 (0.5 - 3.4)	*0.545*	*0.853*
Rosuvastatin		13.8 (8)	5.4 (3)	0.4 (0.1 - 1.3)	*0.141*	*0.634*
Ezetimibe		46.6 (27)	26.8 (15)	0.4 (0.2 - 0.9)	***0.030***	*0.216*
Drug interaction	CYP3A4 inhibitor	13.2 (7)	6.7 (3)	0.5 (0.1 - 1.8)	*0.295*	*1.000*
SRAE		29.3 (17)	3.6 (2)	0.1 (0 - 0.3)	***0.002***	***0.036***
Myopathy		34.5 (20)	7.3 (4)	0.1 (0 - 0.4)	***0.001***	***0.036***
Reduced adherence		17.2 (10)	14.5 (8)	0.8 (0.3 - 2.2)	*0.696*	*0.895*
**Patient characteristics**						
Age		53.3 ± 14.9	56.7 ± 13.9	1.0 (0.99 - 1.04)	*0.215*	*0.645*
Male gender		31.0 (18)	25.0 (14)	0.7 (0.3 - 1.7)	*0.474*	*0.853*
Ethnics	Brown + black	46.6 (27)	58.9 (33)	1.5 (0.7 - 3.4)	*0.288*	*0.741*
BMI (kg/cm²)		26.9 ± 3.5	29.2 ± 5.0	1.1 (1.04 - 1.26)	***0.009***	***0.081***
Type 2 diabetes		25.9 (15)	16.7 (9)	0.6 (0.2 - 1.4)	*0.220*	*0.660*
FH-related variant	Carrier	34.5 (20)	26.8 (15)	0.7 (0.3 - 1.5)	*0.374*	*0.792*

Number of patients in round brackets. Categorical variables are expressed as percentage and number between brackets. Continuous variables are expressed as mean and standard deviation. P-value was adjusted using the Benjamini-Hochberg correction with a FDR of 10%. NRE: non-responder; RE: responder; OR: odds ratio; CI: confidence interval; BMI: body mass index; FH: familial hypercholesterolemia; LDL-c: low-density lipoprotein cholesterol; NR: not reported (No patients in NRE group); SRAE: statin-related adverse events.

Table S14 Association of deleterious variants (MAF> 1.0%) in PK-related genes with statin response in FH patients: Multivariate logistic regression analysis.

**Variable**		**RE**, % (58)	**NRE**, % (56)	**OR (95%CI)**	*p-value*
CYP2C19 c.431G>A	A allele	2.2 (1)	6.4 (3)	2.58 (0.30 - 54.78)	*0.428*
CYP2C8 c.486C>G	A allele	4.4 (2)	12.8 (6)	1.81 (0.33 - 14.3)	*0.520*
CYP2C8 g.5932A>G	G allele	44.4 (20)	42.6 (20)	0.87 (0.33 - 2.26)	*0.772*
*CYP2C9*2* c.430C>T	T allele	12.1 (7)	21.4 (12)	1.65 (0.48 - 6.14)	*0.437*
*CYP2C9*9* c.752A>G	G allele	5.2 (3)	3.6 (2)	4.49 (0.38 - 56.46)	*0.225*
CYP2D6 c.941G>A	A allele	11.1 (5)	8.5 (4)	0.41 (0.07 - 2.1)	*0.287*
CYP2D6 c.100C>T	T allele	11.1 (5)	10.6 (5)	0.4 (0.08 - 1.99)	*0.260*
CYP2D6 g.6866G>A	A allele	2.2 (1)	6.4 (3)	1.09 (0.1 - 25.79)	*0.948*
CYP3A5 c.624G>A	A allele	3.4 (2)	3.6 (2)	0.95 (0.08 - 12.82)	*0.968*
CYP3A5 c.423A>G	G allele	10.3 (6)	12.5 (7)	0.99 (0.25 - 4.01)	*0.986*
*CYP3A5*3* g.12083G>A	A allele	93.1 (54)	94.6 (53)	0.86 (0.11 - 6.38)	*0.881*
UGT1A3 c.808A>G	G allele	4.4 (2)	2.1 (1)	0.13 (0 - 1.95)	*0.166*
ABCC1 c.2012G>T	T allele	15.6 (7)	0.0 (0)	NR	-
ABCC2 c.3872C>T	T allele	2.2 (1)	10.6 (5)	7.58 (0.81 - 199.03)	*0.123*
ABCC2 c.3542G>T	T allele	4.4 (2)	6.4 (3)	1.15 (0.13 - 12.74)	*0.903*
ABCC3 c.3890G>A	A allele	13.3 (6)	12.8 (6)	0.95 (0.24 - 3.96)	*0.946*
SLC15A1 c.364G>A	A allele	4.4 (2)	2.1 (1)	0.1 (0 - 1.33)	*0.098*
SLC22A1 c.181C>T	T allele	4.4 (2)	8.5 (4)	1.34 (0.19 - 12.57)	*0.776*
SLC22A1 c.113G>A	A allele	6.7 (3)	2.1 (1)	0.44 (0.02 - 4.02)	*0.504*
SLC22A1 c.1260_1262del	Deletion	31.1 (14)	38.3 (18)	0.88 (0.31 - 2.5)	*0.813*
*SLCO1B1*5* c.521T>C	C allele	24.1 (14)	19.6 (11)	0.62 (0.2 - 1.84)	*0.391*
SLCO1B1 c.1463G>C	C allele	3.4 (2)	3.6 (2)	1.75 (0.12 - 22.02)	*0.657*
SLCO1B3 c.767G>C	C allele	26.7 (12)	23.4 (11)	0.62 (0.18 - 1.98)	*0.418*

Each model was adjusted with the following covariates: body mass index, baseline LDL-c, therapy intensity, and presence of SRAE. Number of patients in round brackets. NRE: non-responder; RE: responder; OR: odds ratio; CI: confidence interval; FH: familial hypercholesterolemia; NR: not reported (no patients in NRE group); PK: pharmacokinetics; SRAE: statin-related adverse events.

Table S15 Association of variants in PK-related genes and non-genetic variables with SRAE in FH patients: Univariate logistic regression analysis.

**Variant**		**No SRAE (n= 89)**	**SRAE (n=24)**	**OR (95%CI)**	***p*-value**	***Adjusted p-value***
**Deleterious variants**						
CYP2C8 c.486C>G	A allele	45.5 (35)	35.7 (5)	0.7 (0.2 - 2.1)	*0.501*	*0.895*
*CYP2C9*2* c.430C>T	T allele	16.9 (15)	12.5 (3)	0.7 (0.2 - 2.4)	*0.606*	*0.947*
*CYP2C9*3* c.752A>G	G allele	2.2 (2)	12.5 (3)	6.2 (1 - 49.5)	*0.053*	*0.331*
CYP3A5 c.624G>A	A allele	3.4 (3)	4.2 (1)	1.2 (0.1 - 10.3)	*0.852*	*0.926*
CYP3A5 c.423A>G	G allele	11.2 (10)	12.5 (3)	1.1 (0.2 - 4.1)	*0.863*	*0.932*
*CYP3A5*3* g.12083G>A	A allele	93.3 (83)	95.8 (23)	1.7 (0.3 - 32.2)	*0.646*	*0.950*
ABCC1 c.2012G>T	T allele	6.5 (5)	14.3 (2)	2.4 (0.3 - 12.6)	*0.327*	*0.743*
ABCC2 c.3872C>T	T allele	5.2 (4)	14.3 (2)	3.0 (0.4 - 17.5)	*0.227*	*0.568*
ABCC2 c.3542G>T	T allele	5.2 (4)	7.1 (1)	1.4 (0.1 - 10.5)	*0.770*	*1.000*
ABCC3 c.3890G>A	A allele	13 (10)	14.3 (2)	1.1 (0.2 - 5)	*0.895*	*0.932*
SLC22A1 c.113G>A	A allele	3.9 (3)	7.1 (1)	1.9 (0.1 - 16.2)	*0.591*	*0.985*
SLC22A1 c.1260_1262del	Deletion	37.7 (29)	14.3 (2)	0.3 (0 - 1.1)	*0.107*	*0.446*
*SLCO1B1*5* c.521T>C	C allele	21.3 (19)	25 (6)	1.2 (0.4 - 3.4)	*0.702*	*0.975*
SLCO1B1 c.1463G>C	C allele	3.4 (3)	4.2 (1)	1.2 (0.1 - 10.3)	*0.852*	*0.968*
SLCO1B3 c.767G>C	C allele	26 (20)	14.3 (2)	0.5 (0.1 - 1.9)	*0.356*	*0.742*
**Treatment**						
Baseline LDL-c (mg/dL)		240 ± 75	296 ± 90	1 (1.00 - 1.01)	***0.004***	***0.033****
High intensity treatment		84.3 (75)	91.7 (22)	2.1 (0.5 - 13.7)	*0.365*	*0.702*
Atorvastatin		83.1 (74)	66.7 (16)	0.4 (0.1 - 1.2)	*0.081*	*0.405*
Rosuvastatin		7.9 (7)	16.7 (4)	2.3 (0.6 - 8.6)	*0.207*	*0.575*
Ezetimibe		32.6 (29)	50 (12)	2.1 (0.8 - 5.2)	*0.119*	*0.425*
Drug interaction	CYP3A4 inhibitor	5.6 (5)	20.8 (5)	4.4 (1.1 - 17.4)	***0.029***	*0.196*
Reduced adherence		10.1 (9)	37.5 (9)	5.3 (1.8 - 16)	***0.002***	*0.050*
**Patient characteristics**						
Age		55.1 ± 14.4	55.9 ± 13.6	1 (0.97- 1.03)	*0.822*	*1.027*
Gender	Male	27 (24)	29.2 (7)	1.1 (0.4 - 2.9)	*0.830*	*0.988*
Ethnics	Brown + Black	46.1 (35)	45.8 (11)	1.0 (0.4 - 2.5)	*0.985*	*0.985*
BMI		28.4 ± 4.7	26.8 ± 3.4	0.9 (0.81 - 1.02)	*0.134*	*0.419*
FH-related variant	Carrier	22.5 (20)	54.2 (13)	4.1 (1.6 - 10.7)	***0.004***	*0.050*

Number of patients in round brackets. P-value was adjusted using the Benjamini-Hochberg correction. NRE: non-responder; RE: responder; OR: odds ratio; CI: confidence interval; BMI: body mass index; FH: familial hypercholesterolemia; LDL-c: low-density lipoprotein cholesterol; NR: not reported (No patients in NRE group); SRAE: statin-related adverse events.
